# Identification and characterization of TMV-induced volatile signals in *Nicotiana benthamiana*: evidence for JA/ET defense pathway priming in congeneric neighbors via airborne (E)-2-octenal

**DOI:** 10.1007/s10142-023-01203-z

**Published:** 2023-08-12

**Authors:** Yi Hong, Qingxia Zheng, Lingtong Cheng, Pingping Liu, Guoyun Xu, Hui Zhang, Peijian Cao, Huina Zhou

**Affiliations:** 1 China Tobacco Gene Research Center, Zhengzhou Tobacco Research Institute of CNTC, Zhengzhou, 450001 China; 2 Beijing Life Science Academy, Beijing, 102200 China

**Keywords:** (E)-2-Octenal, *Nicotiana benthamiana*, TMV, Plant–plant interaction, JA/ET pathway, Airborne signal

## Abstract

Plants release a mixture of volatile compounds when subjects to environmental stress, allowing them to transmit information to neighboring plants. Here, we find that *Nicotiana benthamiana* plants infected with tobacco mosaic virus (TMV) induces defense responses in neighboring congeners. Analytical screening of volatiles from *N. benthamiana* at 7 days post inoculation (dpi) using an optimized SPME–GC–MS method showed that TMV triggers the release of several volatiles, such as (E)-2-octenal, 6-methyl-5-hepten-2-one, and geranylacetone. Exposure to (E)-2-octenal enhances the resistance of *N. benthamiana* plants to TMV and triggers the immune system with upregulation of pathogenesis-related genes, such as *NbPR1a*, *NbPR1b*, *NbPR2*, and *NbNPR1*, which are related to TMV resistance. Furthermore, (E)-2-octenal upregulates jasmonic acid (JA) that levels up to 400-fold in recipient *N. benthamiana* plants and significantly affects the expression pattern of key genes in the JA/ET signaling pathway, such as *NbMYC2*, *NbERF1*, and *NbPDF1.2*, while the salicylic acid (SA) level is not significantly affected. Our results show for the first time that the volatile (E)-2-octenal primes the JA/ET pathway and then activates immune responses, ultimately leading to enhanced TMV resistance in adjacent *N. benthamiana* plants. These findings provide new insights into the role of airborne compounds in virus-induced interplant interactions.

**Supplementary Information:**

The online version contains supplementary material available at 10.1007/s10142-023-01203-z.

## Introduction

Plant-to-plant (PTP) communication is a way for plants to transmit information to each other (Baldwin and Schultz 1983) and can occur not only between plants of the same species but also between plants of different species (Frank et al. 2021; Markovic et al. 2019; Moreno et al. 2021; Ninkovic et al. 2019; Riedlmeier et al. 2017). Volatile organic compounds (VOCs) released by plants are involved in a wide range of ecological functions and are the medium used for PTP communication (Vivaldo et al. 2017). Plants are sessile organisms that have evolved complex and systematic mechanisms to cope with various stresses, including biotic and abiotic stresses (Greco et al. 2012). When plants perceive stress, they release VOCs, transmitting these airborne signals to nearby plants and thus enhancing the stress resistance of the receiver plant.

Microbe-induced plant volatiles (MIPVs) are a large group of plant VOCs produced as a result of microbial stresses such as bacterial, fungal, and viral infection (Sharifi et al. 2018). A growing number of studies have demonstrated the importance of MIPVs for PTP communication. For instance, *Arabidopsis thaliana* released monoterpenes such as camphene, α-pinene, and β-pinene upon infection with avirulent *Pseudomonas syringae*, and treatment of *Arabidopsis* pants with these volatiles resulted in upregulation of plant reactive oxygen levels as well as expression of disease resistance genes (Adhab 2021; Riedlmeier et al. 2017). Similarly, wheat, a monocotyledonous plant, released ocimene after inoculation with leaf rust pathogen, which caused the expression of the *PR1* gene in neighboring plants and enhanced the disease resistance of the plants (Castelyn et al. 2015). Once plants are infected by viruses, their volatile emissions change (Chang et al. 2021), which in turn affects the behaviors of insect vectors (Chang et al. 2023; Finke 2019).

Plants use a variety of immune mechanisms to defend themselves against pathogen invasion (Song et al. 2015), among which systemic acquired resistance (SAR) and induced systemic resistance (ISR) have received much attention as two main modes of systemic immunity against a broad spectrum of microbial pathogens (Vlot et al. 2021). SAR is triggered by biotrophic or hemibiotrophic pathogens (Glazebrook 2005; Pieterse and van Loon 1999; Vlot et al. 2009), and SAR is closely related to salicylic acid (SA) and SAR-related genes including nonexpresser of pathogenesis-related genes 1 (*NPR1*) (Kim et al. 2020) and pathogenesis-related 1 (*PR1*) (Sharma et al. 2013). In contrast, ISR is mainly triggered by beneficial soil microorganisms (Pieterse et al. 2014) and necrotrophic pathogens (Van der Ent et al. 2008), and the specific process of ISR is closely related to the JA/ET pathway (Nie et al. 2017). Overall, SAR and ISR have important roles as tools of systemic immunity for initiating defense responses and preventing secondary infections.

Tobacco mosaic virus (TMV) is a single-stranded RNA virus in the genus *Tobamovirus* that specifically infects plants, especially tobacco and other Solanaceae plants; it is named for its ability to make infected leaves appear mottled and stained (Scholthof 2004). TMV particles are straight rods, 300 nm × 18 nm in size, and are highly pathogenic and resistant (Stubbs 1999). The virus can survive in dry tobacco leaves for 52 years and remains active even after 1 million-fold dilution (M. et al. 2021). Plants infected with TMV cannot be treated and need to be removed to reduce the damage caused by virus infection in the field; thus, TMV poses a major threat to crop production and food security. Considering the enhancement of disease resistance by pathogen-induced plant volatiles, they have potential applications in the development of new prevention and control strategies for pathogen management, including TMV management. To date, TMV-induced volatiles have not been reported.

In this study, we verified the volatiles of *Nicotiana benthamiana* plants induced by TMV and identified three airborne signals that can transmit warnings to neighboring cogener plants resulting in enhanced TMV resistance. Among these three volatiles, (E)-2-octenal was identified as the most efficient volatile, improving TMV resistance in *N. benthamiana* through activation of the JA/ET pathway.

## Materials and methods

### *Plant materials and growth conditions*

*N. benthamiana* was sown in a plastic pot covered by a layer of cling film and grown for 10 days in a greenhouse under a cycle consisting of 16 h of light at 28 °C and 8 h of darkness at 24 °C. Seedlings were transplanted into new plastic pots individually for further growth under the same conditions. Five-week-old plants were used for TMV inoculation and exposure experiments, and at least three replicates were taken until the time point indicated.

### TMV inoculation and propagation detection

A fresh viral inoculum was prepared from systemically infected leaves of *N. benthamiana* infiltrated with TMV maintained in our laboratory, for which 100 mg of infected leaves was homogenized in 1 mL of 50 mM sodium phosphate buffer (pH 7.0). For TMV inoculation, 5 μL of inoculum was added to the leaves of *N. benthamiana* with fine quartz sand, and the entire leaf was gently rubbed by hand. The mock group was obtained by the same method except that an equal volume of buffer solution was used instead of viral inoculum. The control group was *N. benthamiana* plants without any treatment. TMV-green fluorescent protein (TMV-GFP) (a gift from Prof. Baulcombe and Prof. Zhu) inoculation was carried out as described for TMV inoculation.

To quantify the TMV propagation level, reverse transcription polymerase chain reaction (RT–PCR) (semiquantitative and quantitative) and GFP imaging were used for TMV- and TMV-GFP-inoculated plants, respectively. RT–PCR was performed by using a Roche Light Cycler 96 (Hoffmann-La Roche Ltd, Basel, Switzerland) with the primers TMV-CP-Forward “CTATTTCTAGTGTCAAATGCACCTA” and TMV-CP-Reverse “CAACAAGCTCGAACTGTCGT,” which were designed based on the gene encoding the TMV coat protein (TMV-CP). For semiquantitative RT–PCR, the reaction procedure was as follows: predenaturation at 94 °C for 5 min, followed by 35 cycles of amplification with denaturation at 94 °C for 30 s, annealing at 56 °C for 30 s, and extension at 72 °C for 30 s, ending with a final extension at 72 °C for 7 min. The reaction mixture had a volume of 25 μL, containing 12.5 μL of 2×Taq MasterMix (Hoffmann-La Roche Ltd, Basel, Switzerland), 0.5 μL of each forward and reverse primer, 0.5 μL of cDNA template, and 11 μL of ddH_2_O. The PCR products were detected by 1% agarose gel electrophoresis. For quantitative RT–PCR (qRT–PCR), the reaction volume was 20 μL, including 10 μL of 2×SYBR I Master (Hoffmann-La Roche Ltd, Basel, Switzerland), 0.5 μL of each of the TMV-CP Forward and TMV-CP-Reverse primers, 1 μL of cDNA template, and 8 μL of ddH_2_O to bring the volume up to 20 μL. The PCR procedure was as follows: predenaturation at 94 °C for 30 s, followed by 45 cycles of amplification with denaturation at 94 °C for 5 s, annealing at 60 °C for 10 s, and extension at 72 °C for 10 s. *NbGAPDH* was used as a reference gene (Liu et al. 2012). The relative expression of TMV-CP was calculated using the 2^-ΔΔCt^ method. For GFP imaging, TMV-GFP-infected *N. benthamiana* plants were placed in a dark room and photographed under a 488 nm UV lamp (Adhab et al. 2019).

### RNA isolation, library preparation, and transcriptomic analysis

Total RNA was isolated using TRIzol reagent (Invitrogen) according to the manufacturer’s instructions. Poly (A) + mRNA was purified with oligo (dT) beads, and then, the mRNA was randomly segmented into small fragments by divalent cations in fragmentation buffer (Illumina) at 94 °C for 5 min. These short fragments were used as templates to synthesize first-strand cDNA using random hexamer primers. Second-strand cDNA was synthesized using RNaseH and DNA polymerase I. Short cDNA fragments were purified with a QiaQuick PCR extraction kit (Qiagen). The cDNA fragments were then connected with sequencing adapters according to an Illumina protocol. After agarose gel electrophoresis, target fragments of 300 to 500 bp were selected for PCR amplification to create the final cDNA library. Library cleanup was accomplished using magnetic beads (LB00V60, BGI) before sequencing. Sequencing was performed on an MGISEQ-2000 (MGI Tech Co., Ltd, Shenzhen, China) with 2*150 bp (Lang et al. 2021).

The raw data obtained from sequencing were filtered using the filtering software SOAPnuke (v1.5.2) to remove the reads that contained connectors, those with unknown base (N) content greater than 10%, and those with low quality (with bases with a quality value less than 15 accounting for more than 50% of the total number of bases in the reads). The clean data were then aligned to the reference genome sequence (https://solgenomics.net/ftp/genomes/Nicotiana_benthamiana/assemblies/Niben.genome.v0.4) using HISAT (v2.1.0) software. The RESM (v1.2.8) software package was used to calculate the gene expression levels for each sample. After obtaining the gene data, functional annotation of genes as well as prediction of transcription factors was performed using KEGG (https://www.genome.jp/kegg/) and GO (http://geneontology.org/).

### Analysis of Nicotiana benthamiana plant VOCs

For analysis of *N. benthamiana* VOCs, a method consisting of solid-phase microextraction (SPME) coupled with gas chromatography–mass spectrometry (GC–MS) was built, and two SPME parameters, SPME fiber composition and extraction time, were optimized (Supplementary Figure S1). The optimized SPME method provided the most detected volatiles and the highest total intensities of detected volatiles using an Agilent 1.10 mm PDMS/DVB SPME Arrow and an extraction time of 1 h at 25 °C. Experimentally, *N. benthamiana* plant was placed in a clean bottomless plastic bottle to collect VOC emissions for 1 h by a SPME Arrow conditioned at 250 °C for 10 min prior to use, and then, the SPME Arrow was placed into the GC–MS inlet for 1 min for desorption of volatiles at 250 °C in splitless mode (Lisanti et al. 2021).

Separation and detection of volatiles were accomplished by a Thermo Scientific TRACE 1310 GC-TSQ8000 (USA) equipped with a Thermo Scientific Trace GOLD TG-1MS GC column (60 m × 0.25 mm × 0.25 μm). The GC conditions were as follows: injection temperature, 240 °C; splitless mode; helium at a flow rate of 1.0 mL/min; spacer purge flow rate of 5.0 mL/min; column temperature held at 40 °C for 2 min and then increased to 60 °C at 4 °C/min, 160 °C at 2 °C/min, and 200 °C at 5 °C/min, holding at this temperature for 2.0 min. The MS conditions were as follows: ion source temperature, 280 °C; transmission line temperature, 240 °C; EI mode, 70 eV; solvent delay, 8.50 min; scan range, 40–450 amu. Mass spectrometry data were deconvoluted and analyzed with AUTOGC–MSDATAANAL software (Zhang et al. 2020). The NIST Library (version 2.3) and authentic standards were used for metabolite identification.

### Plant-to-plant (PTP) interaction and exposure experiments

For detection of PTP interaction, four 5-week-old healthy plants, namely, receiver plants, were evenly placed 10 cm away from the emitter TMV-inoculated plant that was set at the bottom center of a cubic glass-container (50 cm × 50 cm × 50 cm), without direct physical contact between any two plants. The whole compartment was placed in the same greenhouse as the receiver plants. To minimize the possible effect of pot soil and plant roots on the adsorption and release of VOCs, all pots were double-wrapped with aluminum foil and cling film in this experiment. The penultimate leaves from receiver plants were collected on day 0 and day 7 of the experiment for subsequent qRT–PCR analysis.

We used a vessel 50 cm in diameter and approximately 50 cm high with a sealed lid for the exposure experiment, and 3 separate experiments were performed. Briefly, an uncovered Petri dish with a diameter of 3 cm was placed in the bottom center of the glass container, and three 5-week-old plants of *N. benthamiana* were placed evenly and equally spaced at 10 cm away from the Petri dish. One milliliter of selected volatile compounds in the form of standard solution was added to the Petri dish and allowed to evaporate naturally for 6 h. The penultimate leaves of each plant were sampled for further experiments at 0 h, 3 h, and 6 h of exposure.

### Virus resistance experiments

After 6 h of exposure, the unsampled intact plants were immediately used for TMV and TMV-GFP virus inoculation. The infected leaves were sampled at 3 dpi, the systemic leaves were sampled at 7 dpi, and samples were immediately frozen in liquid N_2_ for qRT–PCR. GFP imaging was performed for TMV-GFP-inoculated plants of *N. benthamiana* at 0 dpi and 7 dpi.

### Quantification of transcripts

Total RNA was extracted according to the instructions of the Plant RNA Extraction Kit (Tiangen Biochemical Technology Ltd, Beijing, China). The concentration and purity of RNA were measured by a NanoDrop 2000 (Thermo Fisher Scientific Inc., Beijing, China). The reverse transcribed cDNA was synthesized according to the instructions of the Roche First Strand cDNA Synthesis Kit (Hoffmann-La Roche Ltd, Basel, Switzerland) and diluted 5-fold as a cDNA template, which could be stored at −20 °C if not used immediately. Quantification of the targeted transcripts was performed by qRT–PCR as described in the previous section on TMV inoculation and propagation detection. The primers for the transcripts analyzed in this study are shown in Table 1.

**Table 1:** Primers used for RT–PCR analyses

Gene name	Forward primer	Reverse primer
*NbPR1a*	GATGCCCATAACACAGCTCG	CGAGGTTACAATCTGCAGCC
*NbPR1b*	GACAAGTTGGTGTTGGTCCC	GGCTAGGTTTTCGCCGTAAG
*NbPR2*	ACCATCAGACCAAGATGT	TGGCTAAGAGTGGAAGGT
*NbNPR1*	GATACACGGTGCTGCATGTT	AAGCCTAGTGAGCCTCTTGG
*NbPDF1.2*	ATCTGTCTGGGGAAATGGCA	CATGGTCCCTTGAAACGGTG
*NbERF1*	GGCGAATTTTCCGGGAGACT	GGCTCCGATTTTACTTCGCC
*NbMYC2*	GAGATTAGCTGCTTCGCACTG	GCCCGTAGTCGCACCCATA
*NbWRKY33*	TTATAGATGGAGGAAATACG	TTCTCAATGGGATGTGAAT
*NbACCO*	TGTCATTGGCTTCGTTCT	CTCCACTGCCTCTTTCTC
*NbLRR2*	TGGAAGGGAAGTAGCAGTG	TACAAGGTTTGGATGAGGC
*NbEIN3*	AGTCACCATCTCCTCGTT	GAATTATTTCGGGCTACA
*NbMYB*	AAGTTGTAGACTGAGGTGGCT	TGGAGTGGAGTTCAAGGAT
*NbGAPDH*	AGCTCAAGGGAATTCTCGATG	AACCTTAACCATGTCATCTCCC

### JA, SA, and ACC analysis

For hormone analyses of JA, SA, and ACC (1-aminocyclopropane-1-carboxylate), a method based on an UPLC–ESI–MS/MS system (SCIEX QTRAP 6500) was used. Briefly, hormones were extracted by 1.2 mL of precooled 70% methanol from 50 mg sample powders. The analytical conditions were as follows: Agilent SB-C18 (1.8 μm, 2.1 mm * 100 mm) column, pure water with 0.1% formic acid as mobile phase A, acetonitrile with 0.1% formic acid as mobile phase B, programmed gradient elution from 95% phase A to 5% phase A within 9 min followed by 1 min at 5% A, flow rate of 0.35 mL/min, column temperature of 40 °C, and injection volume of 2 μL.

The ESI source operation parameters were as follows: source temperature, 500 °C; ion spray voltage (IS), 5500 V (positive ion mode)/−4500 V (negative ion mode); ion source gas I (GSI), gas II (GSII), and curtain gas (CUR) set at 50, 60, and 25 psi, respectively; collision-activated dissociation (CAD), high. QQQ scans were acquired via MRM experiments with collision gas (nitrogen) set to medium. The DP (declustering potential) and CE (collision energy) for individual MRM transitions were performed with further DP and CE optimization. A specific set of MRM transitions was monitored for each period according to the metabolites eluted within the period.

### Statistical analysis

Metabolites obtained from SPME–GC–MS were analyzed using SIMCA software (version 14.1, Umetrics, Malmö, Sweden), in which the PLS-DA model was developed and validated. Significance analyses of all data in the experiments were performed in OriginPro (version 9.1, OriginLab, USA) and GraphPad Prism 8 (www.graphpad.com) software.

## Results

### TMV propagation in Nicotiana benthamiana

To directly observe TMV infection and propagation, TMV-GFP was used to inoculate *N. benthamiana*. As shown in Supplementary Figure S2a, in systemic leaves, a punctate fluorescent signal appeared at 5 dpi, and then, the GFP fluorescence spread and strengthened quickly, resulting in almost 80% of the leaf area showing GFP signals at 7 dpi. TMV inoculation showed a similar rate of propagation when the gene expression level of the TMV coat protein was measured. Supplementary Figure S2b indicates that the replication of TMV in systemic leaves increased exponentially from 5 dpi to 7 dpi. Additionally, the relative expression level of TMV-CP displayed a 5-fold increase from 5 to 6 dpi, followed by a marked 40-fold increase from 6 to 7 dpi.

### Transcriptomic changes induced by TMV infection

**Fig. 1:**
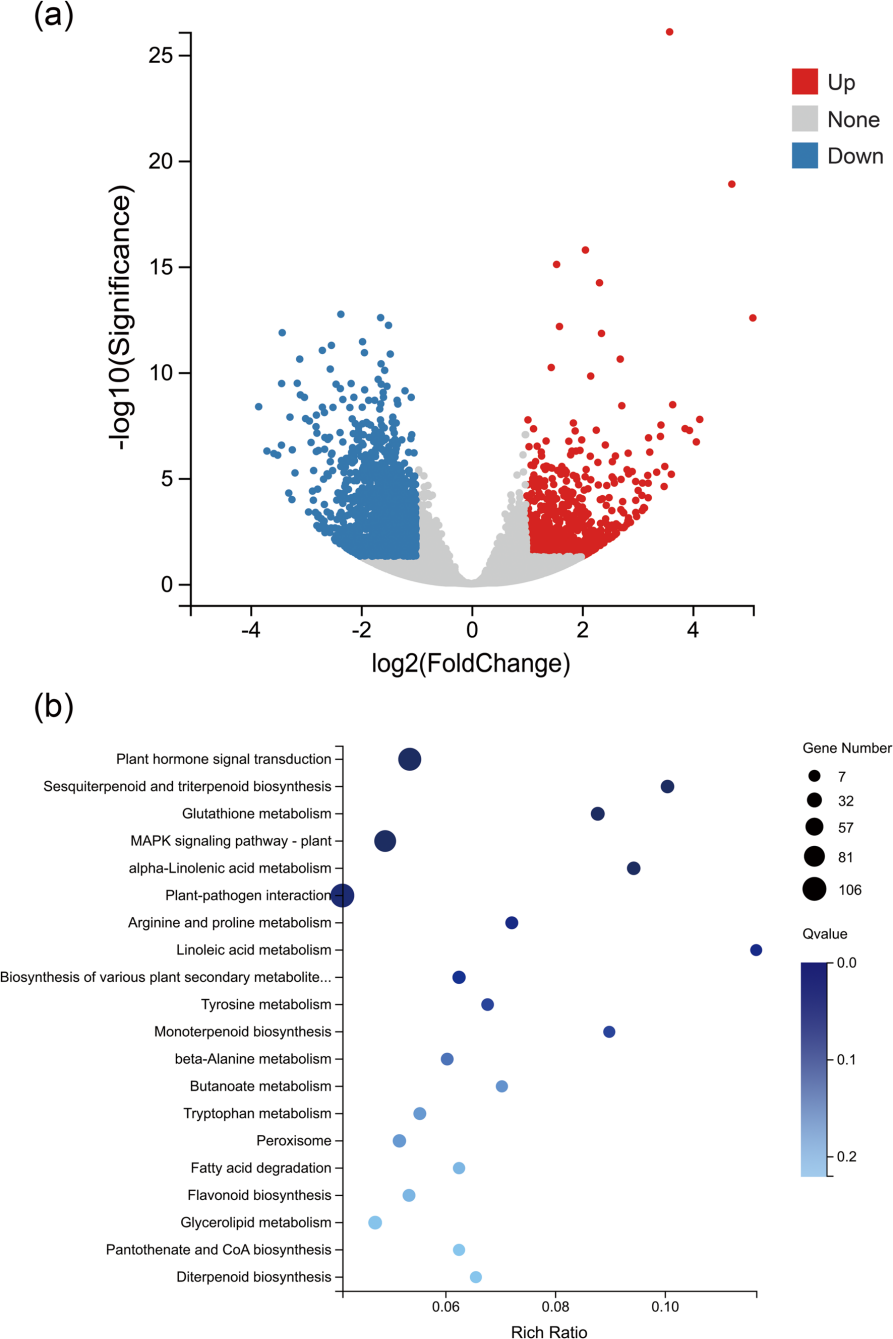
Transcriptomic analyses of systemic leaves from *Nicotiana benthamiana* at 7 days after TMV inoculation. **a** Volcano plot displaying differentially expressed genes (DEGs) of the TMV group compared to the mock group, identified with |log2(fold change) > 1| and *p* value < 0.05. Up, down, and none represent upregulated genes, downregulated genes, and genes with no significant change, respectively. **b** KEGG enrichment analysis of DEGs between the TMV group and mock group. The most enriched KEGG pathways are presented. The horizontal axis represents the rich ratio, while the vertical axis represents the pathway names. Gene number: DEG number; Q value: FDR adjusted *p* value

To gain insight into the responses of *N. benthamiana* to TMV infection at the transcriptome level, at 7 dpi, systemic leaves with high levels of TMV propagation were selected for transcriptome analysis, and the transcriptomic differences between TMV and mock treatments were compared. A total of 5046 differentially expressed genes (DEGs) were identified with |log2(fold change) > 1| and *p* value < 0.05 (Fig. 1a and Supplementary Table S1), of which 1859 genes were upregulated and 3187 genes were downregulated by TMV infection. Among the top 200 DEGs with the greatest fold change (Supplementary Figure S3a), 101 genes were upregulated after TMV infection with log2(fold change) values ranging from 4.98 to 11.72, and 99 genes were downregulated with log2(fold change) values ranging from −4.99 to −9.6, albeit the fold change values of different replicates exhibited some variations. Previously reported TMV resistance-related genes, such as glutathione S-transferase (Gullner et al. 1999; Künstler et al. 2019), *PR1*, and *PR2* (Cornelissen et al. 1986; Wang et al. 2013), were still the most upregulated genes in our study with a total of 101 upregulated genes. The above results demonstrated that *N. benthamiana* underwent a series of alterations in gene expression following TMV infection.

We were particularly intrigued by the upregulated DEGs and their potential roles in conferring resistance to TMV; therefore, we conducted KEGG pathway enrichment analysis on these genes (Supplementary Table S2). As demonstrated in Fig. 1b, the majority of the upregulated DEGs were significantly enriched in pathways such as “Plant–pathogen interaction,” “MAPK signaling pathway – plant,” and “Plant hormone signal transduction,” which are known to be associated with plant disease resistance. Several pathogenesis-related (PR) genes, such as *NbPR1b* and *NbPR1a*, were unsurprisingly enriched in plant–pathogen interaction pathways. These results underscored the importance of these DEGs in conferring protection against TMV infection.

To confirm the accuracy of the transcriptome data, we selected eight upregulated DEGs to conduct qPCR analysis. The results, as presented in Supplementary Figure S3b, showed that all eight genes exhibited higher expression levels after TMV infection, consistent with the findings of the RNA-seq analysis. However, there was a notable difference in the upregulation of *NbEIN3* between the RNA-seq and qPCR results, with the latter showing significantly lower levels of upregulation. Nevertheless, our findings provided strong evidence that the transcriptome data are highly reliable and can be trusted with confidence. According to our transcriptome data, together with reports, we identified *NbPR1b*, *NbPR1a*, and *NbPR2* as genes indicating the initiation of the TMV-induced immune response and the volatile compound-induced immune response.

### Pathogenesis-related (PR) genes in healthy plants were upregulated by PTP communication

**Fig. 2:**
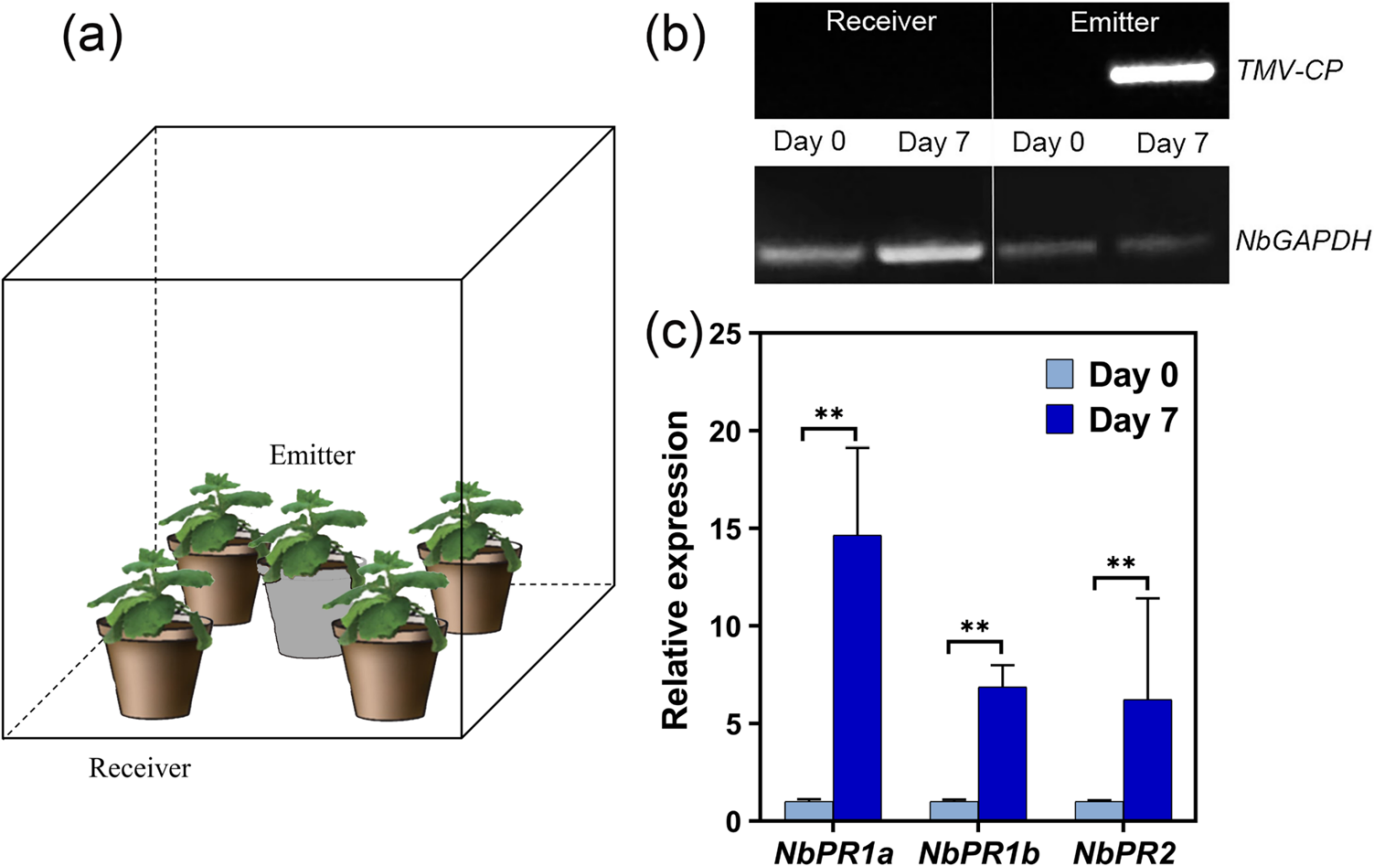
PTP interaction experiment. **a** Schematic diagram illustrating the placement of TMV-infected plants (emitter) and healthy plants (receiver) in the PTP communication experiment. The emitter was placed in the bottom center of the container approximately 10 cm away from its neighbors. All pots in this experiment were double-wrapped with aluminum foil and cling film to minimize the effects of soil absorption and release of VOCs. **b** Semiquantitative RT–PCR analysis of TMV propagation in emitter and receiver plants at day 0 and day 7 of the PTP interaction experiment. The experiment commenced immediately after TMV inoculation of the emitter plant. The products of semiquantitative RT–PCR were analyzed by 1% agarose gel electrophoresis. **c** Quantitative RT–PCR results of the pathogenesis-related gene in receiver plants at day 0 and day 7 of the PTP interaction experiment. Relative expression was calculated using the 2^-ΔΔCt^ method with the reference gene *NbGAPDH*. The data are means ± SDs (*n* = 9). **p < 0.01

To clarify whether there existed airborne signal-mediated communication between the TMV-infected *N. benthamiana* plants and healthy plants nearby but without direct contact, we conducted a PTP interaction experiment by placing healthy plants around the TMV-inoculated plants, as shown in Fig. 2a, and analyzed the expression levels of pathogenesis-related genes and the existence of TMV in healthy plants after 7 days. Figure 2b shows that RNA of the TMV coat protein was detected on inoculated emitter plants at 7 dpi but was not detected in receiver plants, indicating that receiver plants were not infected with TMV. However, the expression levels of pathogenesis-related genes such as *NbPR1a*, *NbPR1b*, and *NbPR2* were significantly upregulated in receiver plants (Fig. 2c), similar to the pattern observed in TMV-infected plants (Supplementary Figure S3b). These results demonstrated that healthy plants received some volatile signal(s) emitted by the TMV-infected plants, which induced immune responses in the healthy plants, preparing them for possible viral invasion in advance.

### VOC profiles of plants are changed by TMV infection

**Fig. 3:**
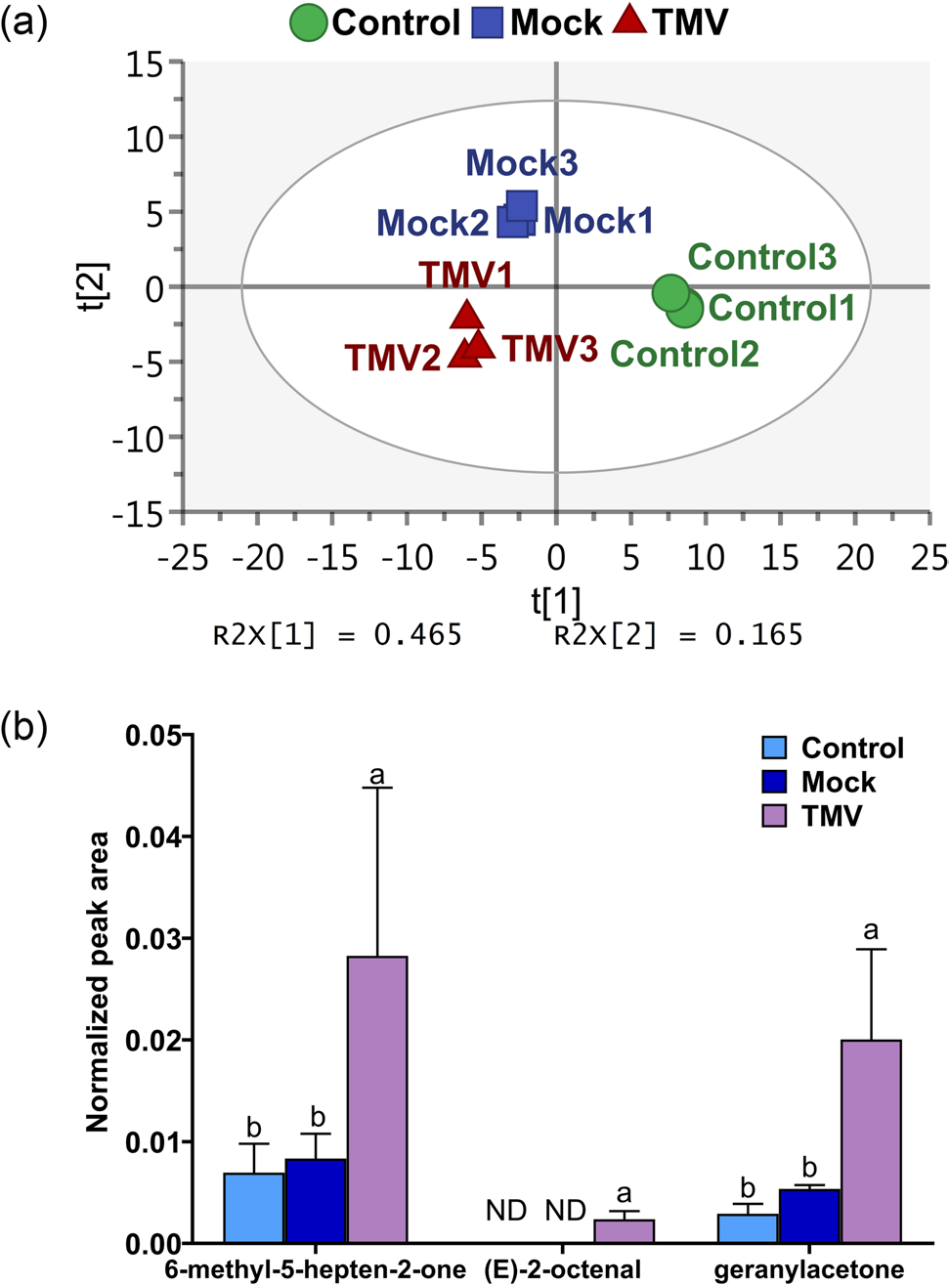
VOC analyses of control, mock, and TMV-infected *Nicotiana benthamiana* plants. **a** PLS-DA score plot showing good separation among the control, mock, and TMV groups. The R2Y (percent of variation in the training set explained by the model) and Q2 (percent of variation in the training set predicted by the model according to cross-validation) of the PLS-DA model were 0.996 and 0.936, respectively, indicating a good model and good predictivity. **b** Release comparison among the control, mock, and TMV groups. (E)-2-Octenal, 6-methyl-5-hepten-2-one, and geranylacetone were specifically upregulated by TMV infection but were not significantly affected by mock treatment. The data are means ± SDs (*n* = 3). ND stands for not detected. Different lowercase letters indicate *p* < 0.05

To investigate the potential role of VOCs in airborne signaling between *N. benthamiana* plants, we analyzed the emissions from TMV-infected plants, mock-treated plants, and control plants at 7 dpi with TMV. Using the SPME–GC–MS method, we identified a total of 93 compounds through NIST library comparison (Supplementary Table S3). To identify VOCs upregulated by TMV infection, we performed a PLS-DA model. The scatter plot (Fig. 3a) indicated well-separated data points for the different treatments. The R2 and Q2 values were both greater than 0.9, indicating that the model had suitable explanatory and predictive power. With a cutoff VIP value greater than 1.0, we identified 23 VOCs that were upregulated in TMV-infected plants, with detection in all 3 replicates (Table 2). Of these, 11 compounds were significantly and specifically induced by TMV infection, of which 4 compounds were only detectable in TMV-infected plants. Of the compounds detected in all three groups, compound 7, 1-ethyl-3-methyl-benzene exhibited the highest increase after TMV treatment, which was 10-fold more than that in the control and mock groups.

**Table 2:** The 23 upregulated VOCs in TMV-infected plants with VIP values greater than 1.0 and detected in all 3 TMV replicates

Compound	Rt (min)	Name	Control	Mock	TMV	VIP
1	10.85	Hexanal	0.00227 ± 0.00020^a^	0.00087 ± 0.00108^a^	0.00334 ± 0.00209^a^	1.23
2	15.66	Heptanal	0.00210 ± 0.00048^b^	0.00427 ± 0.00015^a^	0.00477 ± 0.00057^a^	1.07
3	20.12	Hexahydrofarnesol	0.00009 ± 0.00005^b^	0.00034 ± 0.00009^b^	0.00066 ± 0.00024^a^	1.16
4	20.72	6-Methyl-5-hepten-2-one	0.00695 ± 0.00285^b^	0.00833 ± 0.00244^b^	0.02827 ± 0.01652^a^	1.23
5	21.81	Octanal	0.00733 ± 0.00099^b^	0.01199 ± 0.00028^a^	0.01486 ± 0.00239^a^	1.10
6	23.34	2,3-Dimethyl-cyclohexa-1,3-diene	0.00094 ± 0.00012^a^	0.00122 ± 0.00030^a^	0.00326 ± 0.00253^a^	1.01
7	23.48	1-Ethyl-3-methyl-benzene	0.00093 ± 0.00022^b^	0.00056 ± 0.00014^b^	0.00935 ± 0.00618^a^	1.34
8	24.40	Eucalyptol	0.00037 ± 0.00021^b^	0.00011 ± 0.00003^b^	0.00069 ± 0.00013^a^	1.62
9	25.25	(E)-2-Octenal	ND^b^	ND^b^	0.00236 ± 0.00082^a^	1.53
10	26.04	Glyceryl linolenate	ND^b^	ND^b^	0.00042 ± 0.00012^a^	1.56
11	26.79	Pentylcyclopropane	0.00069 ± 0.00009^b^	0.00133 ± 0.00017^a^	0.00145 ± 0.00015^a^	1.06
12	27.62	(E)-1-Phenyl-1-butene	0.00241 ± 0.00200^a^	0.00205 ± 0.00214^a^	0.00471 ± 0.00039^a^	1.10
13	28.71	Nonanal	0.01910 ± 0.00035^b^	0.02947 ± 0.00163^a^	0.03439 ± 0.00479^a^	1.08
14	29.16	(Hydroxyimino)-3-phenylpropanoic acid	0.00137 ± 0.00044^b^	0.00103 ± 0.00005^b^	0.00739 ± 0.00085^a^	1.63
15	35.79	Decanal	0.02127 ± 0.00112^b^	0.05477 ± 0.00632^a^	0.06693 ± 0.01597^a^	1.05
16	39.38	3-Hydroxydodecanoic acid	0.00105 ± 0.00013^a^	0.00098 ± 0.00007^a^	0.00149 ± 0.00043^a^	1.24
17	40.16	Linoleoyl chloride	0.00109 ± 0.00014^b^	0.00191 ± 0.00031^a^	0.00232 ± 0.00047^a^	1.02
18	42.73	Undecanal	0.00242 ± 0.00012^b^	0.00627 ± 0.00023^a^	0.00680 ± 0.00158^a^	1.04
19	45.28	Methyl arachidonate	0.00102 ± 4.19887^b^	0.00196 ± 0.00016^b^	0.00389 ± 0.00100^a^	1.30
20	49.39	Dodecanal	0.00181 ± 0.00018^b^	0.00407 ± 0.00024^a^	0.00441 ± 0.00077^a^	1.06
21	51.92	Geranylacetone	0.00291 ± 0.00096^b^	0.00535 ± 0.00039^b^	0.02005 ± 0.00886^a^	1.15
22	56.60	(E)-Isovalencenal	ND^b^	ND^b^	0.00362 ± 0.00167^a^	1.40
23	61.14	Geranyl isovalerate	ND^b^	ND^b^	0.00189 ± 0.00124^a^	1.36

ND stands for not detected, and different superscripted lowercase letters indicate a significant difference at a significance level of α = 0.05

To confirm the accuracy of compound identification with the NIST library, we purchased relevant standards and compared their characteristic mass spectra with our results. The identification of three compounds, (E)-2-octenal, 6-methyl-5-hepten-2-one, and geranylacetone, was confirmed to be accurate, as demonstrated in Supplementary Figure S4, and these were used for further analysis. As shown in Table 2 and Fig. 3b, these three VOCs displayed specific upregulation upon TMV infection, as opposed to mock treatment.

### (E)-2-Octenal, 6-methyl-5-hepten-2-one, and geranylacetone enhance the resistance of recipient plants to TMV invasion

**Fig. 4:**
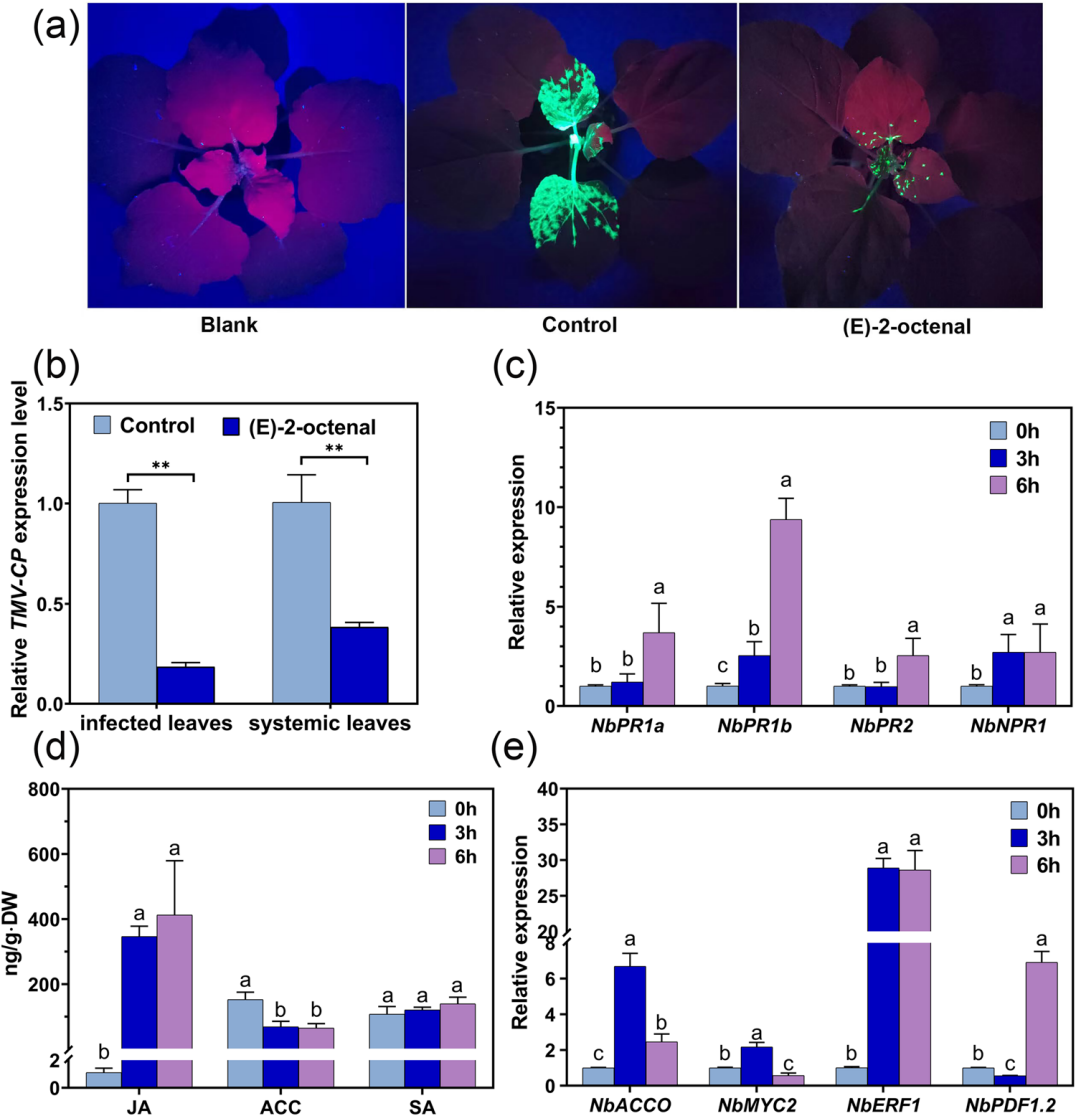
Phenotype, hormone, and gene expression analyses of *Nicotiana benthamiana* plants exposed to (E)-2-octenal. **a** The fluorescence of GFP corresponding to TMV-GFP propagation. Blank represents healthy plants, while control and (E)-2-octenal represent TMV-infected plants at 7 dpi without and with (E)-2-octenal pretreatment, respectively. **b** qRT–PCR analysis of TMV-CP expression. Infected leaves and systemic leaves were sampled at 3 dpi and 7 dpi. **c**, **e** qRT–PCR analysis of genes related to TMV resistance in *Nicotiana benthamiana*. **d** JA, ACC, and SA content changes upon (E)-2-octenal treatment. Relative expression was calculated using the 2^-ΔΔCt^ method with the reference gene *NbGAPDH*. The data are means ± SDs (*n* =3 (**b**, **d**), *n* = 9 (**c**, **e**)). **p < 0.01. Different lowercase letters indicate significance at *p* < 0.05

To verify whether (E)-2-octenal, 6-methyl-5-hepten-2-one, and geranylacetone could be airborne signals that improve the TMV resistance of receiver plants, we conducted exposure experiments. *N. benthamiana* plants and 1 mL of synthetic (E)-2-octenal, 6-methyl-5-hepten-2-one, and geranylacetone were placed in one container for 6 h, followed by inoculation with TMV-GFP immediately after treatment. At 7 dpi, GFP imaging results showed that the TMV-GFP fluorescence area and spot number on leaves of *N. benthamiana* plants from three volatile exposures were all significantly smaller than those of control plants (Supplementary Figure S5a; Fig. 4a). (E)-2-Octenal treatment showed the weakest GFP fluorescence, suggesting that it had the strongest effect in enhancing the resistance of *N. benthamiana* against TMV invasion. Correspondingly, the TMV content in the inoculated leaves at 3 dpi and in systemic leaves at 7 dpi was also significantly lower in plants treated with the three volatiles (Supplementary Figure S5b; Fig. 4b). Among the three compounds, the (E)-2-octenal treatment was the most effective in enhancing TMV resistance; hence, it was used in a subsequent study to discover its possible mechanism of improving *N. benthamiana* resistance to TMV.

### (E)-2-Octenal primes similar TMV defense responses through airborne transmission

To determine whether (E)-2-octenal enhances *N. benthamiana* resistance to TMV by initiating an immune response in the host plant, we examined the expression levels of the immune response-related genes *NbPR1a*, *NbPR1b*, *NbPR2*, and *NbNPR1* in (E)-2-octenal-exposed plants. As shown in Fig. 4c, at 3 h of exposure, the expression levels of *NbPR1b* and *NbNPR1* were significantly upregulated approximately 2.5-fold, while no significant changes were observed for *NbPR1a* and *NbPR2*. When the exposure time was extended to 6 h, all four genes exhibited significant upregulation compared to the level at the start point of 0 h, while *NbPR1b* was continuously upregulated to approximately 9 times the level at 0 h. In contrast, *NbNPR1* remained at a high level without a significant increase from 3 h. Obviously, *NbPR1b* and *NbNPR1* showed more rapid and earlier responses than *NbPR1a* and *NbPR2*. The upregulation of these genes, as key genes of the SAR-related immune response, might suggest that (E)-2-octenal could enhance host plant resistance to TMV by initiating an immune response related to SAR.

### (E)-2-Octenal increases JA levels in recipient plants

To further clarify whether (E)-2-octenal enhances TMV resistance through SA-mediated SAR, we examined the hormone levels of *N. benthamiana* exposed to (E)-2-octenal. As shown in Fig. 4d, the JA content showed a substantial increase, 400 times higher than that at 0 h after 3 h of exposure to (E)-2-octenal, while the content of the ethylene precursor, ACC, exhibited a significant decrease after (E)-2-octenal exposure, more than 50% lower than that at 0 h. Interestingly, the SA content did not demonstrate any significant increase. Notably, these hormone levels did not display a clear response to the duration of treatment. In other words, after 3 h of treatment, these hormone levels had already been regulated into a responsive steady state, indicating a rapid response of hormones to (E)-2-octenal treatment.

We also observed upregulation of the ACC oxidase gene (*NbACCO*). As shown in Fig. 4e, in *N. benthamiana* plants exposed to (E)-2-octenal for 3 h, the expression of the *ACCO* gene increased nearly 7-fold. Even though the magnitude of the increase declined after 6 h, it was still more than twice that at 0 h. ACCO is the key enzyme that directly catalyzes the synthesis of ethylene from ACC. Therefore, it could be reasonable to deduce that ethylene release might also be upregulated by (E)-2-octenal treatment to maintain consistency with the upregulation of *ACCO* and consumption of ACC.

### JA/ET-related defense pathway in healthy plants responding to airborne 2-octenal

Based on the above results, the significant increase in JA, deduced increase in ethylene, and unchanged SA, we hypothesized that the immune response triggered by (E)-2-octenal may be mainly initiated via the JA/ET signaling pathway. To validate this conjecture, we further examined the expression levels of key genes involved in the JA/ET pathway, namely, *NbMYC2*, *NbERF1*, and *NbPDF1.2*. As shown in Fig. 4e, *NbERF1* rapidly and strongly responded to the airborne (E)-2-octenal signal, with its expression level increasing nearly 30-fold and remaining at this high level until 6 h posttreatment. *NbMYC2* expression increased significantly after 3 h of treatment but was markedly reduced at 6 h compared to 0 h. In contrast, *NbPDF1.2* expression displayed an inverse trend with *NbMYC2*, showing a significant decrease at 3 h and a significant increase at 6 h after receiving the (E)-2-octenal signal. Both *MYC2* and *ERF1* can initiate immune responses by regulating the expression of *PDF1.2*. The reported negative regulation of *PDF1.2* by *MYC2* is consistent with our findings. When *NbMYC2* expression is downregulated, the suppression of *PDF1.2* is alleviated, and the high expression of *NbERF1* promotes the elevated expression of *NbPDF1.2*. In summary, (E)-2-octenal treatment triggers a response in the JA/ET signaling pathway, leading to the activation of defense responses.

## Discussion

Plants have evolved mechanisms for interacting with neighbors that depend on changes in the volatiles emitted by plants (Brambilla et al. 2022). This interplant communication plays an important role in improving plant resistance to biotic and abiotic stresses. Previous research has focused on herbivore-induced plant interactions (Jian et al. 2021; Kessler and Baldwin 2004; Schuman et al. 2009), but less on microbe-induced interactions, such as virus-induced, plant interactions (Song et al. 2022). Our study provides the first complete and systematic demonstration that TMV-induced VOCs from *N. benthamiana* can induce defense responses in neighboring plants.

In our study, we analyzed the differences in the transcriptome of *N. benthamiana* before and after virus inoculation, and the 5046 identified DEGs, which were previously identified as TMV resistance-related genes, such as glutathione S-transferase (Gullner et al. 1999; Künstler et al. 2019; Masoodi et al. 2022), *PR1*, and *PR2* (Cornelissen et al. 1986; Wang et al. 2013), were among the most upregulated genes (Fig. 1 and Supplementary Table S1). Thus, three genes associated with SAR, *NbPR1a*, *NbPR1b*, and *NbPR2*, were selected as immune response indicators. Next, we described the upregulation of these three genes in healthy *N. benthamiana* plants after 7 days of cohabitation with TMV-infected plants (Fig. 2). This phenomenon is consistent with previous studies (Bennett 2021; Brosset and Blande 2022; López-Berenguer et al. 2021) and again demonstrates that there is indeed “communication” between plants.

To detect the plant volatiles induced by TMV infection, an SPME–GC–MS analytical method was established, which exhibited good sensitivity and resolution for *N. benthamiana* volatiles and could stably detect 93 volatile compounds from *N. benthamiana*. After statistical analysis and comparison with reference standards, three TMV-specifically induced volatiles were identified, namely, (E)-2-octenal, 6-methyl-5-hepten-2-one, and geranylacetone (Fig. 3 and Supplementary Figure S4). (E)-2-Octenal is widely present as a flavoring substance in a variety of plants (Cheng et al. 2020; Ebert et al. 2022; Raffo et al. 2018), and previous results have shown that plant-produced (E)-2-octenal causes severe perturbation of the lipid fraction of bacterial plasma membranes and inhibits the activity of microorganisms such as rhizospheric fungi and *Candida* (Battinelli et al. 2006; Trombetta et al. 2002). A recent publication (Sdiri et al. 2022) found a significant increase in 6-methyl-5-hepten-2-one levels in olive plants after inoculation with *S*epia *acuminata*, and the authors speculated that 6-methyl-5-hepten-2-one may play a role in pathogen infection. Page et al. (Page et al. 2014) found that flowers of *Silene latifolia* and *Silene dioica* hybrids released 6-methyl-5-hepten-2-one and influenced pollinator preference. Previous studies (Badra et al. 2021; Wróblewska-Kurdyk et al. 2022) have shown that geranylacetone plays an important role in plant-insect interactions. However, the role of these three VOCs in plant-TMV interactions has not been reported.

To investigate the influence of TMV-induced volatiles released from infected *N. benthamiana* plants on neighboring healthy plants, we conducted a virus resistance experiment. Our results showed for the first time that (E)-2-octenal, 6-methyl-5-hepten-2-one, and geranylacetone could enhance the resistance of receiver plants to TMV infection (Supplementary Figure S5; Fig. 4a, b). Furthermore, (E)-2-octenal induced the upregulation of *NbPR1a*, *NbPR1b*, *NbPR2*, and *NbNPR1* in receiver plants (Fig. 4c). Ribnicky et al. (Ribnicky et al. 1998) showed that inoculation with TMV induced the emission of benzaldehyde from *Nicotiana tabacum* which triggered upregulation of the *PR-1* gene in receiver plants and increased plant resistance to TMV. Another study (Shulaev et al. 1997) found that *Nicotiana tabacum* released volatile methyl salicylate (MeSA) upon infection with TMV and that MeSA enhanced the expression of disease resistance genes and resistance to TMV in receiver plants. In our study, we did not find a significant increase in the release of MeSA (Supplementary Table S3 compound 51) as well as benzaldehyde (Supplementary Table S3 compound 11) after TMV inoculation. This may be due to the differences in the response of *N. benthamiana* and *N. tabacum* to TMV or in the method of analysis. In summary, our study identified three TMV-induced plant volatiles that function in airborne signaling, (E)-2-octenal, 6-methyl-5-hepten-2-one, and geranylacetone, which provides new clues for further understanding the mechanism of plant-TMV interactions and may help in the development of new strategies for TMV control.

It has been reported that airborne signals frequently enhance resistance in receiver plants through SAR-induced immune responses (Brambilla et al. 2022; Eccleston et al. 2022; Song and Ryu 2013). SAR is triggered by plant hormones such as SA or its methylated derivative, MeSA. The PTP experiment and exposure experiment revealed that SAR-related genes such as *NbPR1a*, *NbPR1b*, and *NbPR2* were all upregulated (Fig. 2b and Fig. 4c), and we were particularly interested in determining whether (E)-2-octenal activated SAR via SA, which exhibited the most potent resistance to TMV. We analyzed the levels of several hormones in *N. benthamiana* leaves exposed to (E)-2-octenal. No significant influence of (E)-2-octenal on SA levels was observed in the receiver plant, but a significant increase in JA content and a substantial decrease in the ethylene precursor ACC were discovered (Fig. 4d). Given the upregulation of the ACC oxidase gene (*NbACCO*), which directly catalyzes the conversion of ACC to ethylene (Fig. 4e), we hypothesize that the decline in ACC levels occurs due to enhanced ethylene synthesis activity, leading to increased ethylene release. Thus, whether (E)-2-octenal activates SAR in an SA-independent manner requires further study.

The expression levels of key genes in the JA/ET pathway, such as *NbMYC2*, *NbERF1*, and *NbPDF1.2*, showed distinct responses to (E)-2-octenal, with *NbERF1* consistently overexpressed, *NbMYC2* expression initially increasing and then decreasing, and *NbPDF1.2* expression first decreasing and then increasing (Fig. 4e). *NbPDF1.2* expression is negatively regulated by *NbMYC2*. *MYC2*, regulated by JA signaling, primarily activates wounding responses, and the observed upregulation of *NbMYC2* expression after 3 h of (E)-2-octenal treatment is consistent with a prevailing hypothesis that the response mechanism of plants to volatile signals simulates damage-associated signals (Brosset and Blande 2022; Lorenzo et al. 2004). ERF1 is a transcription factor that positively regulates *PDF1.2* in the ethylene signaling pathway, but *NbPDF1.2* is upregulated under positive regulation of *NbERF1* only when *NbMYC2* expression is downregulated. This phenomenon could be due to two reasons. One is that NbMYC2 modulates *NbPDF1.2* expression downstream of NbERF1, and the other is that NbMYC2 may have a stronger regulatory effect on the JA/ET pathway than NbERF1. Godoy et al. (Godoy et al. 2011) suggest that ERF1 binds to DNA with more stringent base requirements than MYC2, which indirectly suggests that MYC2 may have a stronger regulatory function than ERF1.

Generally, the defense response primed by JA/ET is related to ISR. In our study, upregulation of JA levels and the JA/ET pathway was discovered, indicating that (E)-2-octenal may also initiate the activation of ISR in addition to SAR. Previous studies have shown that almost all ISR occurs in the roots of plants. For instance, the work of Wu et al. (Wu et al. 2018) indicated that *Bacillus amyloliquefaciens* realized ISR activation in roots via the JA/ET pathway in *N. benthamiana*. Similarly, Samaras et al. (Samaras et al. 2021) found that the induction of ISR occurred after the application of *Bacillus subtilis* by soil drenching on the roots of tomato plants. However, the ISR triggered by plant volatiles in this study occurred on the foliage of plants. Overall, our study provides new clues about the way plants achieve ISR activation, but the specific mechanisms by which plant volatiles are recognized by the foliage and achieve ISR are still unclear, and these need to be explored in future research.

**Fig. 5:**
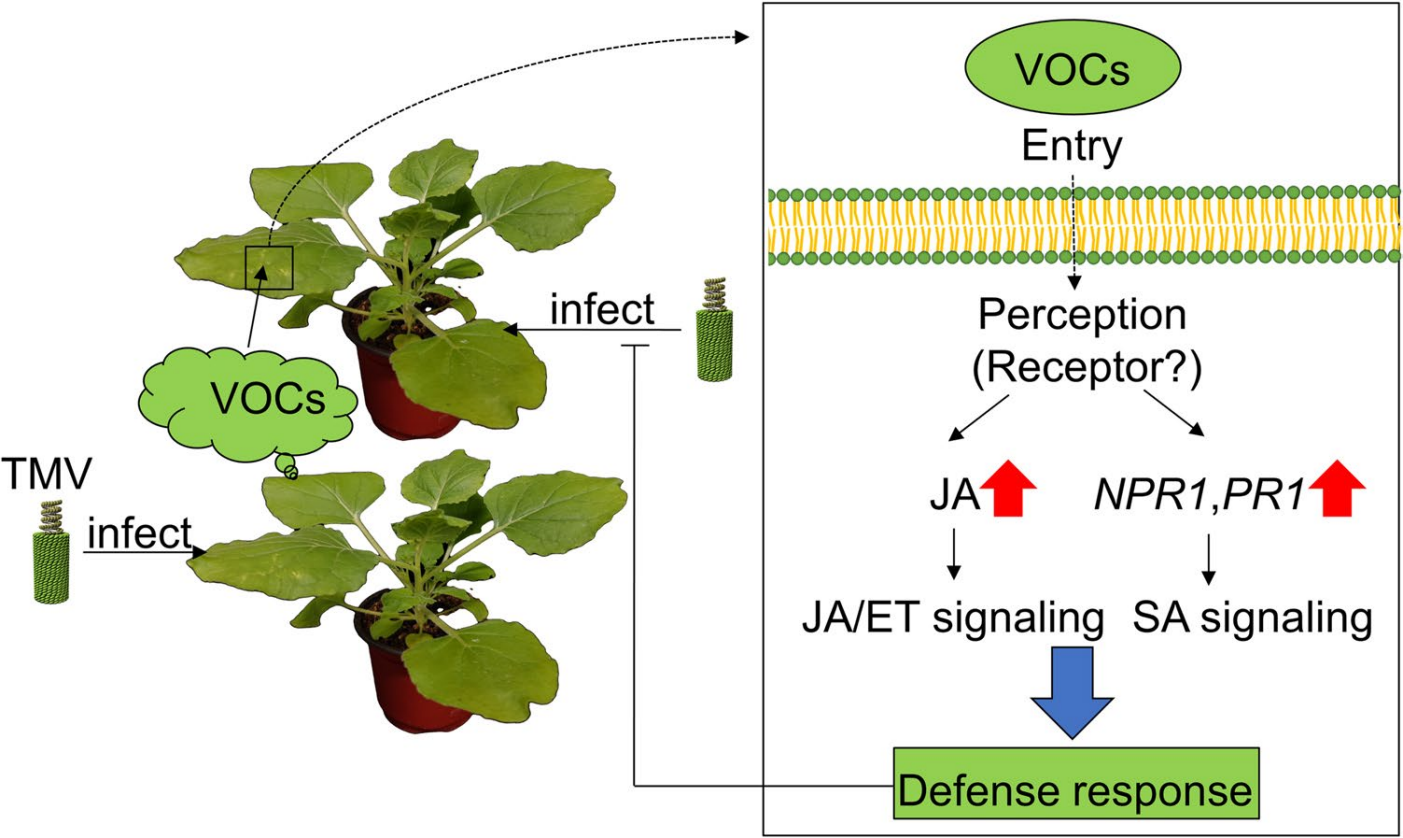
TMV–plant–plant interaction model. TMV-infected *Nicotiana benthamiana* plants emit induced VOCs (airborne signals) such as (E)-2-octenal, which diffuse to nearby neighboring plants. The airborne signals enter the cells by either a to-be-defined way such as stomatal, cuticle, binding, and transporter proteins or by another way to provoke the defense responses. After entry, the signals are perceived by a putative receptor. In our study, (E)-2-octenal, a TMV-induced VOC, strongly induced JA synthesis and the JA/ET signaling pathway. Furthermore, SA signaling-specific PR1 and NPR1 expression was also upregulated, which suggests that SA signaling might also be involved in the (E)-2-octenal-induced defense response in an SA-independent manner, since the SA of *Nicotiana benthamiana* plants was not significantly affected by (E)-2-octenal

Based on this study, we identified the entire mechanism of TMV–plant–plant interactions, as shown in Fig. 5. TMV-infected *N. benthamiana* plants emit induced VOCs (airborne signals) such as (E)-2-octenal, which diffuse to nearby neighboring plants. Then, the airborne signals enter the cells by either a to-be-defined pathway, such as stomatal, cuticle, or odorant-binding protein (OBP)-like transporters (Loreto and D’Auria 2022; Wang and Erb 2022), and are then perceived by a putative receptor, or by other pathways to provoke defense responses. In our study, (E)-2-octenal, a TMV-induced VOC, strongly induced excessively high JA accumulation and the JA/ET signaling pathway. Furthermore, SA signaling-specific PR1 and NPR1 expression was also upregulated, which suggests that SA signaling might also be involved in the (E)-2-octenal-induced defense response in an SA-independent manner since the SA of *N. benthamiana* plants was not significantly affected by (E)-2-octenal exposure treatment. López-Gresa et al. (López-Gresa et al. 2018) reported that a volatile ester, (Z)-3-hexenyl butyrate, could induce PR1 upregulation and stomatal closure against *Pseudomonas syringae* pv. *tomato* in an SA-independent manner. Our results suggested that the (E)-2-octenal-induced defense response may not be related to stomatal closure, since JA is reported to positively regulate the stomatal aperture (Gupta et al. 2020).

In conclusion, we found that TMV triggered the release of (E)-2-octenal, 6-methyl-5-hepten-2-one, and geranylacetone in *N. benthamiana* plants, which activated the immune system of neighboring healthy plants and increased plant disease resistance. Thus, TMV-induced volatiles play an important role in plant–plant interactions. Understanding the interactions between viruses and plants that rely on airborne volatile compounds can help to further understand the mechanism of plant response to virus invasion and would be very important for early warning and forecasting of viral diseases in plants and for green prevention and control.

## Supplementary information

ESM 1

## Data Availability

The RNA-seq datasets presented in this study can be found in online repositories. The address of the repository/repositories and accession number(s) can be found at https://ngdc.cncb.ac.cn/gsa/PRJCA018115.

## References

[CR1] Adhab M (2021). Be smart to survive: virus-host relationships in nature. J Microbiol Biotechnol Food Sci.

[CR2] Adhab M, Angel C, Rodriguez A, Fereidouni M, Király L, Scheets K, Schoelz JE (2019). Tracing the lineage of two traits associated with the coat protein of the Tombusviridae: silencing suppression and HR elicitation in Nicotiana species. Viruses.

[CR3] Badra Z, Larsson Herrera S, Cappellin L, Biasioli F, Dekker T, Angeli S, Tasin M (2021). Species-specific induction of plant volatiles by two aphid species in apple: real time measurement of plant emission and attraction of lacewings in the wind tunnel. J Chem Ecol.

[CR4] Baldwin IT, Schultz JC (1983). Rapid changes in tree leaf chemistry induced by damage: evidence for communication between plants. Science.

[CR5] Battinelli L, Daniele C, Cristiani M, Bisignano G, Saija A, Mazzanti G (2006). In vitro antifungal and anti-elastase activity of some aliphatic aldehydes from Olea europaea L. fruit. Phytomedicine.

[CR6] Bennett T (2021). Plant-plant interactions. Plant Cell Environ.

[CR7] Brambilla A, Sommer A, Ghirardo A, Wenig M, Knappe C, Weber B, Amesmaier M, Lenk M, Schnitzler JP, Vlot AC (2022). Immunity-associated volatile emissions of β-ionone and nonanal propagate defence responses in neighbouring barley plants. J Exp Bot.

[CR8] Brosset A, Blande JD (2022). Volatile-mediated plant-plant interactions: volatile organic compounds as modulators of receiver plant defence, growth, and reproduction. J Exp Bot.

[CR9] Castelyn HD, Appelgryn JJ, Mafa MS, Pretorius ZA, Visser B (2015). Volatiles emitted by leaf rust infected wheat induce a defence response in exposed uninfected wheat seedlings. Australasian Plant Pathology.

[CR10] Chang X, Guo Y, Ren Y, Li Y, Wang F, Ye G, Lu Z (2023). Virus-induced plant volatiles promote virus acquisition and transmission by insect vectors. Int J Mol Sci.

[CR11] Chang X, Wang F, Fang Q, Chen F, Yao H, Gatehouse AMR, Ye G (2021). Virus-induced plant volatiles mediate the olfactory behaviour of its insect vectors. Plant Cell Environ.

[CR12] Cheng G, Chang P, Shen Y, Wu L, El-Sappah AH, Zhang F, Liang Y (2020). Comparing the flavor characteristics of 71 tomato (Solanum lycopersicum) accessions in Central Shaanxi. Front Plant Sci.

[CR13] Cornelissen BJ, Hooft van Huijsduijnen RA, Van Loon LC, Bol JF (1986). Molecular characterization of messenger RNAs for ‘pathogenesis related’ proteins la, lb and lc, induced by TMV infection of tobacco. EMBO J.

[CR14] Ebert S, Michel W, Nedele AK, Baune MC, Terjung N, Zhang Y, Gibis M, Weiss J (2022). Influence of protein extraction and texturization on odor-active compounds of pea proteins. J Sci Food Agric.

[CR15] Eccleston L, Brambilla A, Vlot AC (2022). New molecules in plant defence against pathogens. Essays Biochem.

[CR16] Finke JSMAD (2019). Turnip aphids (Lipaphis erysimi) discriminate host plants based on the strain of cauliflower mosaic virus infection. Emir J Food Agric.

[CR17] Frank L, Wenig M, Ghirardo A, van der Krol A, Vlot AC, Schnitzler JP, Rosenkranz M (2021). Isoprene and β-caryophyllene confer plant resistance via different plant internal signalling pathways. Plant Cell Environ.

[CR18] Glazebrook J (2005). Contrasting mechanisms of defense against biotrophic and necrotrophic pathogens. Annu Rev Phytopathol.

[CR19] Godoy M, Franco-Zorrilla JM, Pérez-Pérez J, Oliveros JC, Lorenzo O, Solano R (2011). Improved protein-binding microarrays for the identification of DNA-binding specificities of transcription factors. Plant J.

[CR20] Greco M, Chiappetta A, Bruno L, Bitonti MB (2012). In Posidonia oceanica cadmium induces changes in DNA methylation and chromatin patterning. J Exp Bot.

[CR21] Gullner G, Tóbiás I, Fodor J, Kömives T (1999). Elevation of glutathione level and activation of glutathione-related enzymes affect virus infection in tobacco. Free Radic Res.

[CR22] Gupta A, Bhardwaj M, Tran LP (2020). Jasmonic acid at the crossroads of plant immunity and Pseudomonas syringae virulence. Int J Mol Sci.

[CR23] Jian G, Jia Y, Li J, Zhou X, Liao Y, Dai G, Zhou Y, Tang J, Zeng L (2021). Elucidation of the regular emission mechanism of volatile β-ocimene with anti-insect function from tea plants (Camellia sinensis) exposed to herbivore attack. J Agric Food Chem.

[CR24] Kessler A, Baldwin IT (2004). Herbivore-induced plant vaccination. Part I. The orchestration of plant defenses in nature and their fitness consequences in the wild tobacco Nicotiana attenuata. Plant J.

[CR25] Kim Y, Gilmour SJ, Chao L, Park S, Thomashow MF (2020). Arabidopsis CAMTA transcription factors regulate pipecolic acid biosynthesis and priming of immunity genes. Mol Plant.

[CR26] Künstler A, Király L, Kátay G, Enyedi AJ, Gullner G (2019). Glutathione can compensate for salicylic acid deficiency in tobacco to maintain resistance to tobacco mosaic virus. Front Plant Sci.

[CR27] Lang J, Zhu R, Sun X, Zhu S, Li T, Shi X, Sun Y, Yang Z, Wang W, Bing P, He B, Tian G (2021) Evaluation of the MGISEQ-2000 sequencing platform for illumina target capture sequencing libraries. 12:730519. 10.3389/fgene.2021.730519 10.3389/fgene.2021.730519 PMC8578046 34777467

[CR28] Lisanti MT, Laboyrie J, Marchand-Marion S, de Revel G, Moio L, Riquier L, Franc C (2021). Minty aroma compounds in red wine: development of a novel automated HS-SPME-arrow and gas chromatography-tandem mass spectrometry quantification method. Food Chem.

[CR29] Liu D, Shi L, Han C, Yu J, Li D, Zhang Y (2012). Validation of reference genes for gene expression studies in virus-infected Nicotiana benthamiana using quantitative real-time PCR. PloS One.

[CR30] López-Berenguer C, Donaire L, González-Ibeas D, Gómez-Aix C, Truniger V, Pechar GS, Aranda MA (2021). Virus-infected melon plants emit volatiles that induce gene deregulation in neighboring healthy plants. Phytopathology.

[CR31] López-Gresa MP, Payá C, Ozáez M, Rodrigo I, Conejero V, Klee H, Bellés JM, Lisón P (2018). A new role for green leaf volatile esters in tomato stomatal defense against Pseudomonas syringe pv. tomato. Front Plant Sci.

[CR32] Lorenzo O, Chico JM, Sánchez-Serrano JJ, Solano R (2004). JASMONATE-INSENSITIVE1 encodes a MYC transcription factor essential to discriminate between different jasmonate-regulated defense responses in Arabidopsis. Plant Cell.

[CR33] Loreto F, D’Auria S (2022). How do plants sense volatiles sent by other plants?. Trends Plant Sci.

[CR34] White JC, Hao Y, He Z, Rui Y, M. A, T. F (2021). Carbon-based nanomaterials suppress tobacco mosaic virus (TMV) infection and induce resistance in Nicotiana benthamiana. J Hazard Mater.

[CR35] Markovic D, Colzi I, Taiti C, Ray S, Scalone R, Gregory Ali J, Mancuso S, Ninkovic V (2019). Airborne signals synchronize the defenses of neighboring plants in response to touch. J Exp Bot.

[CR36] Masoodi KZ, Ahmed N, Mir MA, Bhat B, Shafi A, Mansoor S, Rasool RS, Yaseen M, Dar ZA, Mir JI, Andrabi SM, Ganai NA (2022). Comparative transcriptomics unravels new genes imparting scab resistance in apple (Malus x domestica Borkh.). Funct Integr Genomics.

[CR37] Moreno JC, Mi J, Alagoz Y, Al-Babili S (2021). Plant apocarotenoids: from retrograde signaling to interspecific communication. Plant J.

[CR38] Nie P, Li X, Wang S, Guo J, Zhao H, Niu D (2017). Induced systemic resistance against Botrytis cinerea by Bacillus cereus AR156 through a JA/ET- and NPR1-dependent signaling pathway and activates PAMP-triggered immunity in Arabidopsis. Front Plant Sci.

[CR39] Ninkovic V, Rensing M, Dahlin I, Markovic D (2019). Who is my neighbor? Volatile cues in plant interactions. Plant Signal Behav.

[CR40] Page P, Favre A, Schiestl FP, Karrenberg S (2014). Do flower color and floral scent of silene species affect host preference of Hadena bicruris, a seed-eating pollinator, under field conditions?. PloS One.

[CR41] Pieterse CM, van Loon LC (1999). Salicylic acid-independent plant defence pathways. Trends Plant Sci.

[CR42] Pieterse CM, Zamioudis C, Berendsen RL, Weller DM, Van Wees SC, Bakker PA (2014). Induced systemic resistance by beneficial microbes. Annu Rev Phytopathol.

[CR43] Raffo A, Masci M, Moneta E, Nicoli S, Sánchez Del Pulgar J, Paoletti F (2018). Characterization of volatiles and identification of odor-active compounds of rocket leaves. Food Chem.

[CR44] Ribnicky DM, Shulaev V, Raskin I (1998). Intermediates of salicylic acid biosynthesis in tobacco. Plant Physiol.

[CR45] Riedlmeier M, Ghirardo A, Wenig M, Knappe C, Koch K, Georgii E, Dey S, Parker JE, Schnitzler JP, Vlot AC (2017). Monoterpenes support systemic acquired resistance within and between plants. Plant Cell.

[CR46] Samaras A, Roumeliotis E, Ntasiou P, Karaoglanidis G (2021). Bacillus subtilis MBI600 promotes growth of tomato plants and induces systemic resistance contributing to the control of soilborne pathogens. Plants (Basel).

[CR47] Scholthof KB (2004). Tobacco mosaic virus: a model system for plant biology. Annu Rev Phytopathol.

[CR48] Schuman MC, Heinzel N, Gaquerel E, Svatos A, Baldwin IT (2009). Polymorphism in jasmonate signaling partially accounts for the variety of volatiles produced by Nicotiana attenuata plants in a native population. New Phytol.

[CR49] Sdiri Y, Lopes T, Rodrigues N, Silva K, Rodrigues I, Pereira JA, Baptista P (2022). Biocontrol ability and production of volatile organic compounds as a potential mechanism of action of olive endophytes against Colletotrichum acutatum. Microorganisms.

[CR50] Sharifi R, Lee SM, Ryu CM (2018). Microbe-induced plant volatiles. New Phytol.

[CR51] Sharma R, De Vleesschauwer D, Sharma MK, Ronald PC (2013). Recent advances in dissecting stress-regulatory crosstalk in rice. Mol Plant.

[CR52] Shulaev V, Silverman P, Raskin I (1997). Airborne signalling by methyl salicylate in plant pathogen resistance. Nature.

[CR53] Song GC, Choi HK, Ryu CM (2015). Gaseous 3-pentanol primes plant immunity against a bacterial speck pathogen, Pseudomonas syringae pv. tomato via salicylic acid and jasmonic acid-dependent signaling pathways in Arabidopsis. Front Plant Sci.

[CR54] Song GC, Jeon JS, Choi HK, Sim HJ, Kim SG, Ryu CM (2022). Bacterial type III effector-induced plant C8 volatiles elicit antibacterial immunity in heterospecific neighbouring plants via airborne signalling. Plant Cell Environ.

[CR55] Song GC, Ryu CM (2013). Two volatile organic compounds trigger plant self-defense against a bacterial pathogen and a sucking insect in cucumber under open field conditions. Int J Mol Sci.

[CR56] Stubbs G (1999). Tobacco mosaic virus particle structure and the initiation of disassembly. Philos Trans R Soc Lond B Biol Sci.

[CR57] Trombetta D, Saija A, Bisignano G, Arena S, Caruso S, Mazzanti G, Uccella N, Castelli F (2002). Study on the mechanisms of the antibacterial action of some plant alpha,beta-unsaturated aldehydes. Lett Appl Microbiol.

[CR58] Van der Ent S, Verhagen BW, Van Doorn R, Bakker D, Verlaan MG, Pel MJ, Joosten RG, Proveniers MC, Van Loon LC, Ton J, Pieterse CM (2008). MYB72 is required in early signaling steps of rhizobacteria-induced systemic resistance in Arabidopsis. Plant Physiol.

[CR59] Vivaldo G, Masi E, Taiti C, Caldarelli G, Mancuso S (2017). The network of plants volatile organic compounds. Sci Rep.

[CR60] Vlot AC, Dempsey DA, Klessig DF (2009). Salicylic acid, a multifaceted hormone to combat disease. Annu Rev Phytopathol.

[CR61] Vlot AC, Sales JH, Lenk M, Bauer K, Brambilla A, Sommer A, Chen Y, Wenig M, Nayem S (2021). Systemic propagation of immunity in plants. New Phytol.

[CR62] Wang J, Wang HY, Xia XM, Li PP, Wang KY (2013). Inhibitory effect of esterified lactoferin and lactoferrin against tobacco mosaic virus (TMV) in tobacco seedlings. Pestic Biochem Physiol.

[CR63] Wang L, Erb M (2022). Volatile uptake, transport, perception, and signaling shape a plant’s nose. Essays Biochem.

[CR64] Wróblewska-Kurdyk A, Dancewicz K, Gliszczyńska A, Gabryś B (2022). Antifeedant potential of geranylacetone and nerylacetone and their epoxy-derivatives against Myzus persicae (Sulz.). Molecules.

[CR65] Wu L, Huang Z, Li X, Ma L, Gu Q, Wu H, Liu J, Borriss R, Wu Z, Gao X (2018). Stomatal closure and SA-, JA/ET-signaling pathways are essential for Bacillus amyloliquefaciens FZB42 to restrict leaf disease caused by Phytophthora nicotianae in Nicotiana benthamiana. Front Microbiol.

[CR66] Zhang YY, Zhang Q, Zhang YM, Wang WW, Zhang L, Yu YJ, Bai CC, Guo JZ, Fu HY, She Y (2020). A comprehensive automatic data analysis strategy for gas chromatography-mass spectrometry based untargeted metabolomics. J Chromatogr A.

