# Supplementary figures and images for: Identification and characterization of TMV-induced volatile signals in Nicotiana benthamiana: evidence for JA/ET defense pathway priming in congeneric neighbors via airborne (E)-2-octenal
Source: Funct Integr Genomics. 2023 Aug 12;23(3):272. doi: 10.1007/s10142-023-01203-z (PMC10421810; doi:10.1007/s10142-023-01203-z)

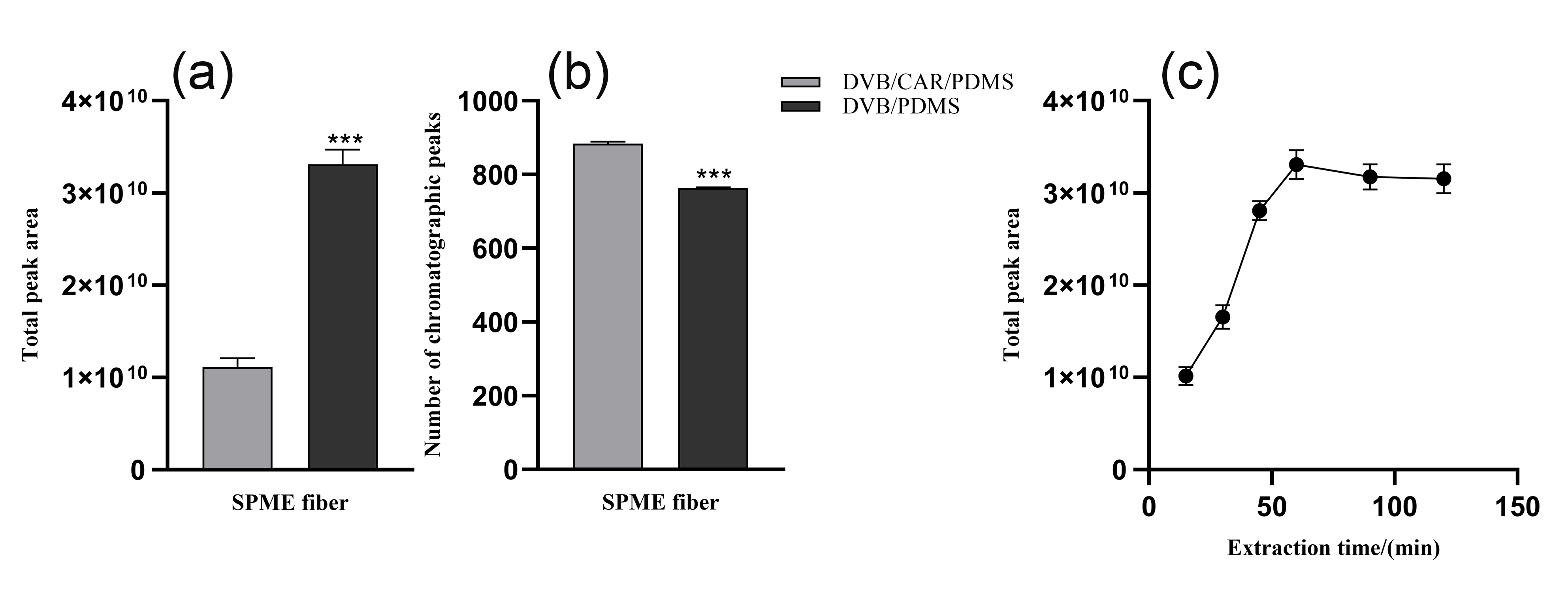

Supplement: Supplementary file 1 — ESM 1 [file 10142_2023_1203_MOESM1_ESM.zip › Supplementary Figure S1.tif]

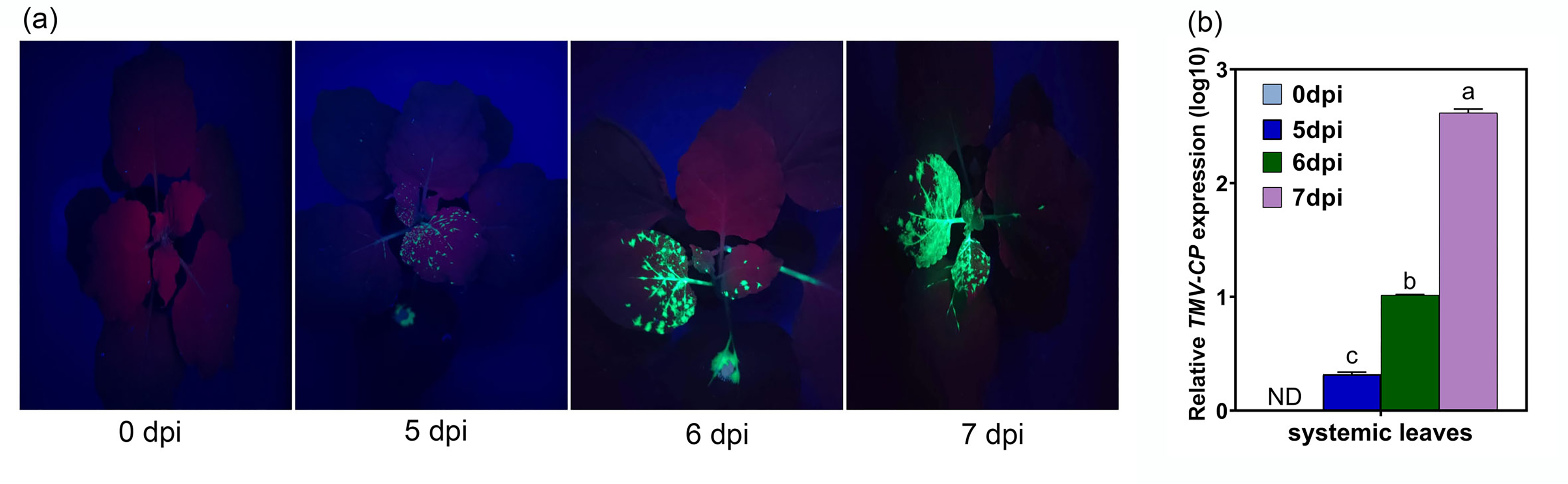

Supplement: Supplementary file 1 — ESM 1 [file 10142_2023_1203_MOESM1_ESM.zip › Supplementary Figure S2.tif]

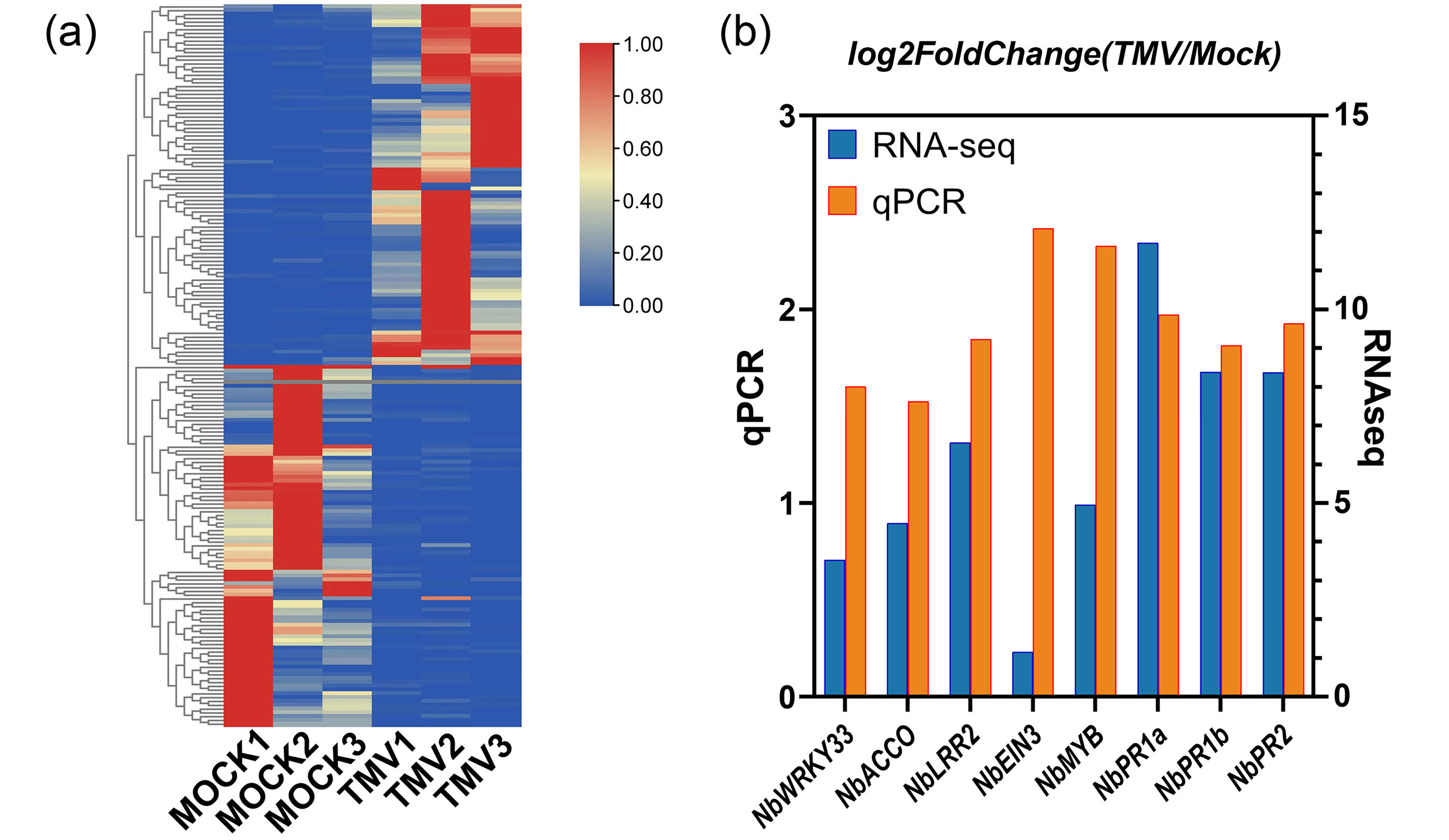

Supplement: Supplementary file 1 — ESM 1 [file 10142_2023_1203_MOESM1_ESM.zip › Supplementary Figure S3.tif]

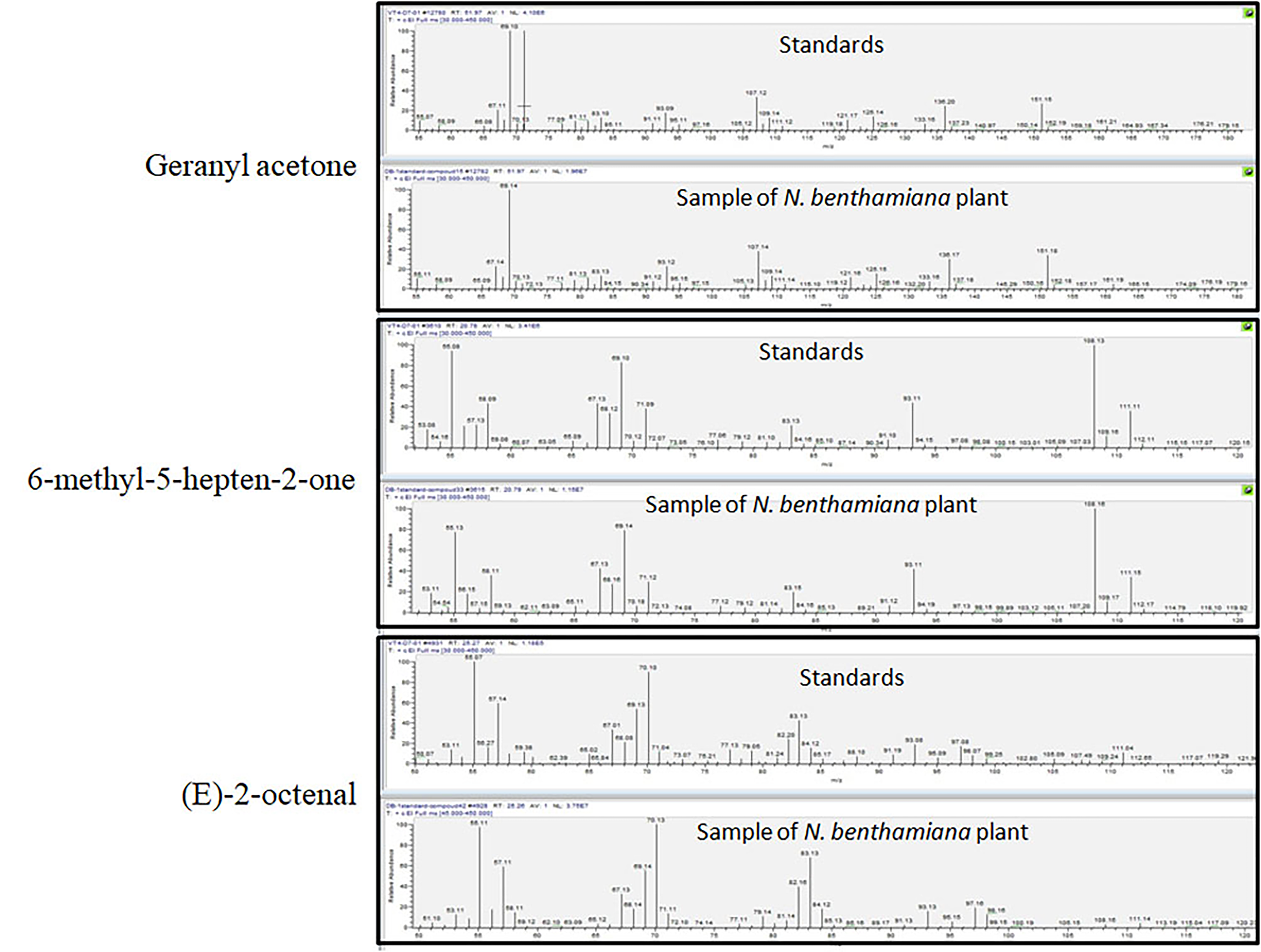

Supplement: Supplementary file 1 — ESM 1 [file 10142_2023_1203_MOESM1_ESM.zip › Supplementary Figure S4.tif]

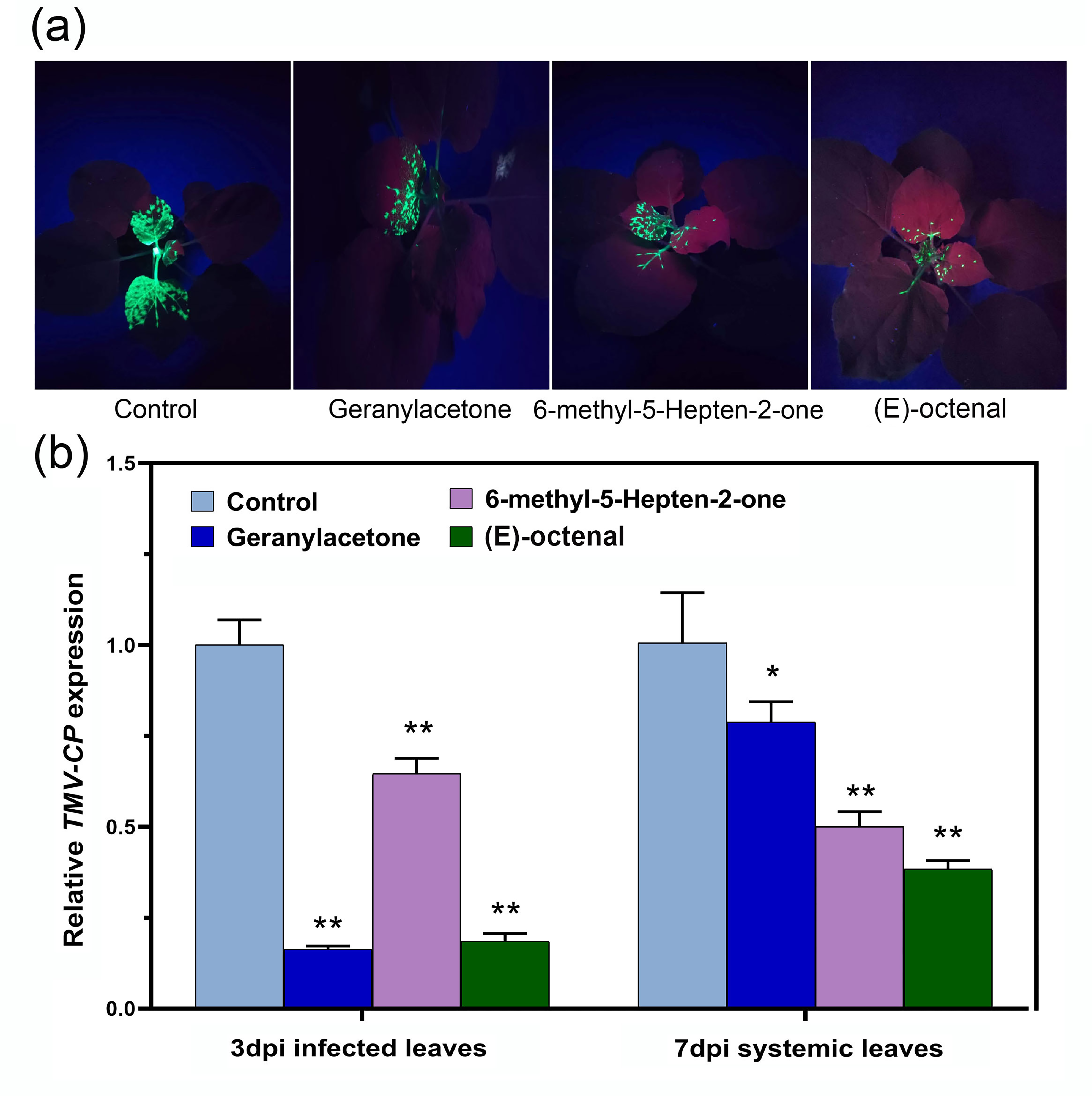

Supplement: Supplementary file 1 — ESM 1 [file 10142_2023_1203_MOESM1_ESM.zip › Supplementary Figure S5.tif]
